# Supplementary material for: Association between oral microbiome and seven types of cancers in East Asian population: a two-sample Mendelian randomization analysis
Source: Front Mol Biosci. 2023 Nov 21;10:1327893. doi: 10.3389/fmolb.2023.1327893 (PMC10702768; doi:10.3389/fmolb.2023.1327893)
Supplement: Supplementary file 1 [file DataSheet1.ZIP › Supplementary Materials/MR plots for tongue/tongue═╝/Pancreatic cancer/╥╚╧┘░⌐_╔╕╤í╜ß╣√/pheno.1762_to_pancreatic cancer_leave_one_out.pdf]

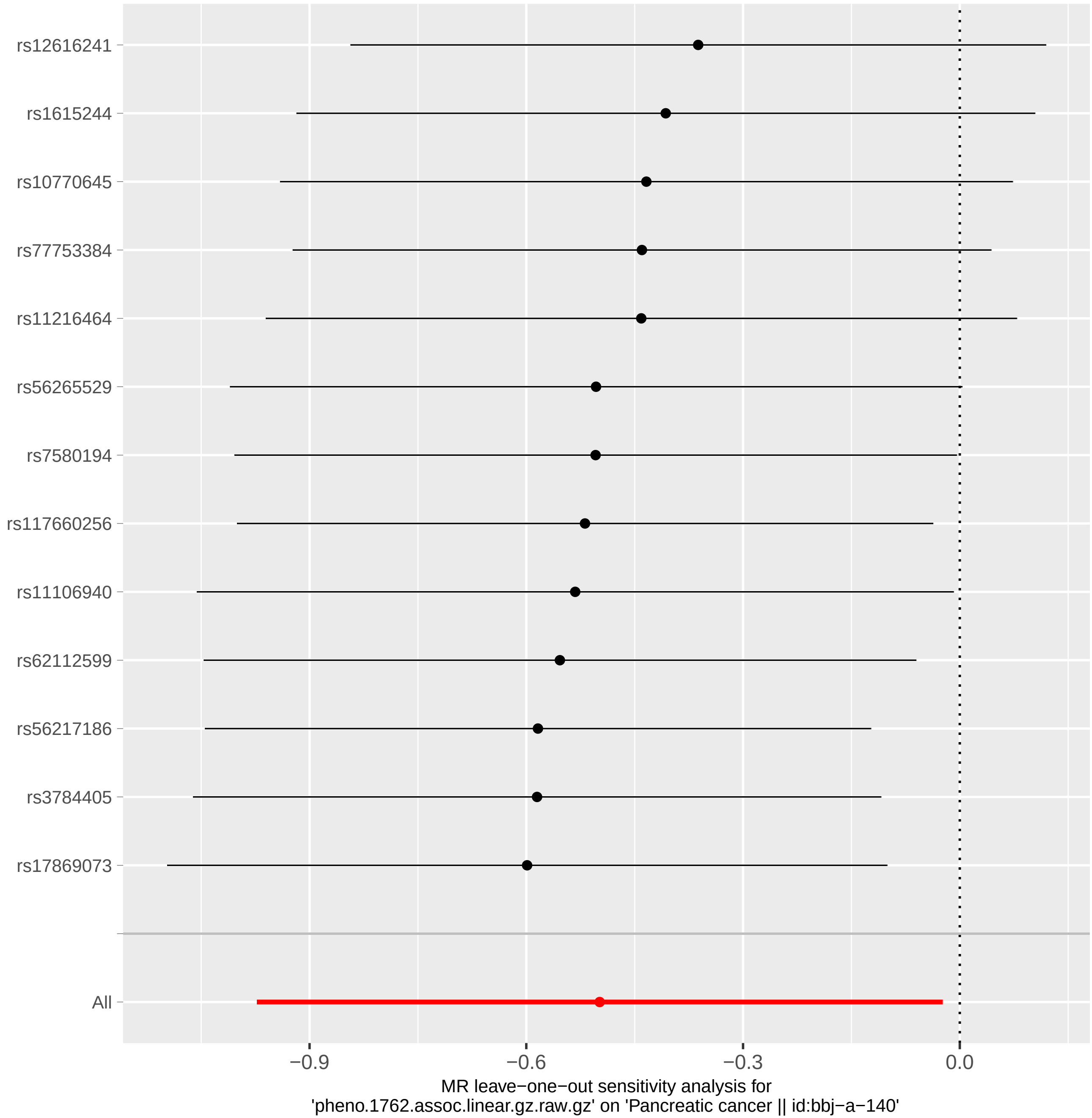
rs12616241
rs1615244
rs10770645
rs77753384
rs11216464
rs56265529
rs7580194
rs117660256
rs11106940
rs62112599
rs56217186
rs3784405
rs17869073
All
−0.9
−0.6
−0.3
0.0
MR leave−one−out sensitivity analysis for
'pheno.1762.assoc.linear.gz.raw.gz' on 'Pancreatic cancer || id:bbj−a−140'
